# Shedding light on biodiversity: reviewing existing knowledge and exploring hypothesised impacts of agrophotovoltaics

**DOI:** 10.1111/brv.13165

**Published:** 2024-11-10

**Authors:** Rachel Schwarz, Yaron Ziv

**Affiliations:** ^1^ Spatial Ecology Lab, Department of Life Sciences Ben‐Gurion University of the Negev P.O.B. 653 Beer‐Sheva 84105 Israel; ^2^ Leibniz Institute of Freshwater Ecology and Inland Fisheries (IGB) Müggelseedamm 310 Berlin 12587 Germany; ^3^ Institute of Biology Freie Universität Berlin Königin‐Luise‐Str. 1‐3 Berlin 14195 Germany

**Keywords:** agroecology, conservation, ecological processes, ecosystem services, environmental impact

## Abstract

The growing demand for energy and the shift towards green energy solutions have led to the conversion of open spaces and agricultural fields into photovoltaic (PV) power plants, exacerbating the “food–energy–environment” trilemma. Agrophotovoltaics (APVs), a dual‐use system combining agriculture and energy production on the same land, presents a potential solution to this challenge. While the environmental impacts of ground‐mounted utility‐scale PV (USPV) power plants and the effects of APV systems on agricultural yields have been extensively studied and reviewed, the implications for wildlife and biodiversity remain largely unexplored. This knowledge gap is pressing, given the accelerated global adoption of APV systems and the urgency of understanding their broader ecological consequences. In this concise review, we synthesise existing literature on the impacts of USPV installations on biodiversity and the effects of APV on crop production. Building on these foundations, we propose novel hypotheses concerning the potential pathways and mechanisms through which APV systems may influence biodiversity. We explore the complex interactions between agroecosystems and natural ecosystems, examining both direct and indirect effects. Our review culminates in a set of key research questions designed to guide future studies on the biodiversity outcomes of APV deployment. Future research should comprehensively address factors such as habitat type, climate, spatial scale, technology, and agricultural practices, as well as the overarching impacts of climate change. By highlighting the importance of these variables, we aim to facilitate a nuanced understanding of how APV systems can either support or undermine biodiversity. This work not only underscores the critical need for empirical studies in this emerging field but also sets the stage for more informed and sustainable implementation of APV technologies.

## I. INTRODUCTION

Habitat destruction and global warming are nowadays recognised as the two main drivers of the biodiversity crisis worldwide (Sala *et al*., 2000; Newbold *et al*., 2015; IPCC, 2022). The growing human population increases food demand, leading to the conversion of natural habitats into agricultural fields (Vitousek *et al*., 1997; EEA, 2011; Lambin & Meyfroidt, 2011). Currently croplands and pastures cover about 50% of the global land surface (Foley *et al*., 2005). Energy demands are also rising, and considering climate change, investment in renewable energy, especially solar power, is increasing (EEA, 2011). Solar photovoltaics (PV) is the fastest‐growing renewable energy technology (IEA, 2022). Most large PV plants are utility‐scale [>1 megawatts (MW)], ground‐mounted facilities (USPV; Turney & Fthenakis, 2011; Solar Power Europe, 2018).

The demand and subsidies for renewable energy, along with the abandonment of agricultural land due, for example, to low profitability and climate change (Rey Benayas *et al*., 2007), often leads to the conversion of arable and natural land into power plants (Tilman *et al*., 2009; Baffes & Haniotis, 2010; EEA, 2011; Rosillo‐Calle, 2012; Hartmann *et al*., 2016; Hernandez *et al*., 2019; Walston *et al*., 2021). This competition for land is known as the “food–energy–environment” trilemma (Tilman *et al*., 2009). One solution that has been advocated since first conceptualised by Goetzberger & Zastrow (1982) is using the same land area for agriculture and solar‐energy production, that is dual use (Dupraz *et al*., 2011; Barron‐Gafford *et al*., 2019). The coexistence of crop cultivation or animal husbandry and the installation of solar photovoltaic modules on the same land is known as agrovoltaics or agrophotovoltaics (APV). In some cases, APV can increase land productivity by up to 70% and achieve higher land‐equivalent ratios [a quantitative metric of the reduction in land use (Weselek *et al*., 2019; Nordberg, Caley & Schwarzkopf, 2021)]. This technology, therefore, has the potential to prevent the destruction and conversion of additional natural habitats (Hernandez *et al*., 2019; Nordberg *et al*., 2021), as well as reduce the competition for land between food and energy production (Elborg, 2017).

Open APV systems can be either high‐mounted, installed on stilts 2–5 m above ground (e.g. Marrou, Dufour & Wery, 2013a; Marrou *et al*., 2013, 2013), or ground‐mounted, with increased spacing between PV arrays (e.g. Trommsdorff *et al*., 2021; de la Torre *et al*., 2022). Each system has benefits and drawbacks, but they share similar characteristics, such as casting shade that changes the microclimate directly beneath and around the PV panels (Weselek *et al*., 2019). While some microclimatic differences may reduce crop production in certain areas, in others, advantages such as additional shading and water conservation can improve crop production (Barron‐Gafford *et al*., 2019; Hernandez *et al*., 2019; Weselek *et al*., 2019, 2021). The microclimate under the PV panels in APV fields may not only affect the crops grown underneath, but may also impact other co‐occurring plants and animals, such as pollinators, natural enemies, pests and pathogens, and invasive species (Graham *et al*., 2021). Additional impacts of PV panels in agricultural fields may include glare, electromagnetic fields, polarised light emissions, the introduction of novel physical structures, and effects from installation and maintenance processes (Tsoutsos, Frantzeskaki & Gekas, 2005; Chiabrando, Fabrizio & Garnero, 2009; Spellman, 2014; Rabaia *et al*., 2021).

The effect of APV systems is mainly local, affecting wild species co‐occurring in the APV fields. However, regional effects, such as spillover of impact from the APV ecosystem into the natural ecosystem, can occur. Such effects can create wide‐ranging, large‐scale changes in the surrounding ecosystem. To date, much research has been done on the impacts of APV systems on agricultural yield (e.g. Barron‐Gafford *et al*., 2019; Weselek *et al*., 2019; Sarr *et al*., 2023), and the environmental effects of ground‐mounted USPV power plants (e.g. Tsoutsos *et al*., 2005; Chiabrando *et al*., 2009; Turney & Fthenakis, 2011; Hernandez *et al*., 2014; Spellman, 2014; Sánchez‐Zapata *et al*., 2016; Rabaia *et al*., 2021). However, studies on the effects of PV panels on biodiversity (see review by Lafitte *et al*., 2023) and ecosystem services (e.g. Armstrong *et al*., 2014; de Marco et al., 2014; Armstrong, Ostle & Whitaker, 2016; Randle‐Boggis *et al*., 2020) are fewer, and research on the environmental effects of APV systems and their impact on biodiversity is even rarer (Kagan *et al*., 2014; Walston *et al*., 2016; Sinha *et al*., 2018; Graham *et al*., 2021; Nordberg *et al*., 2021).

**Fig. 1:**
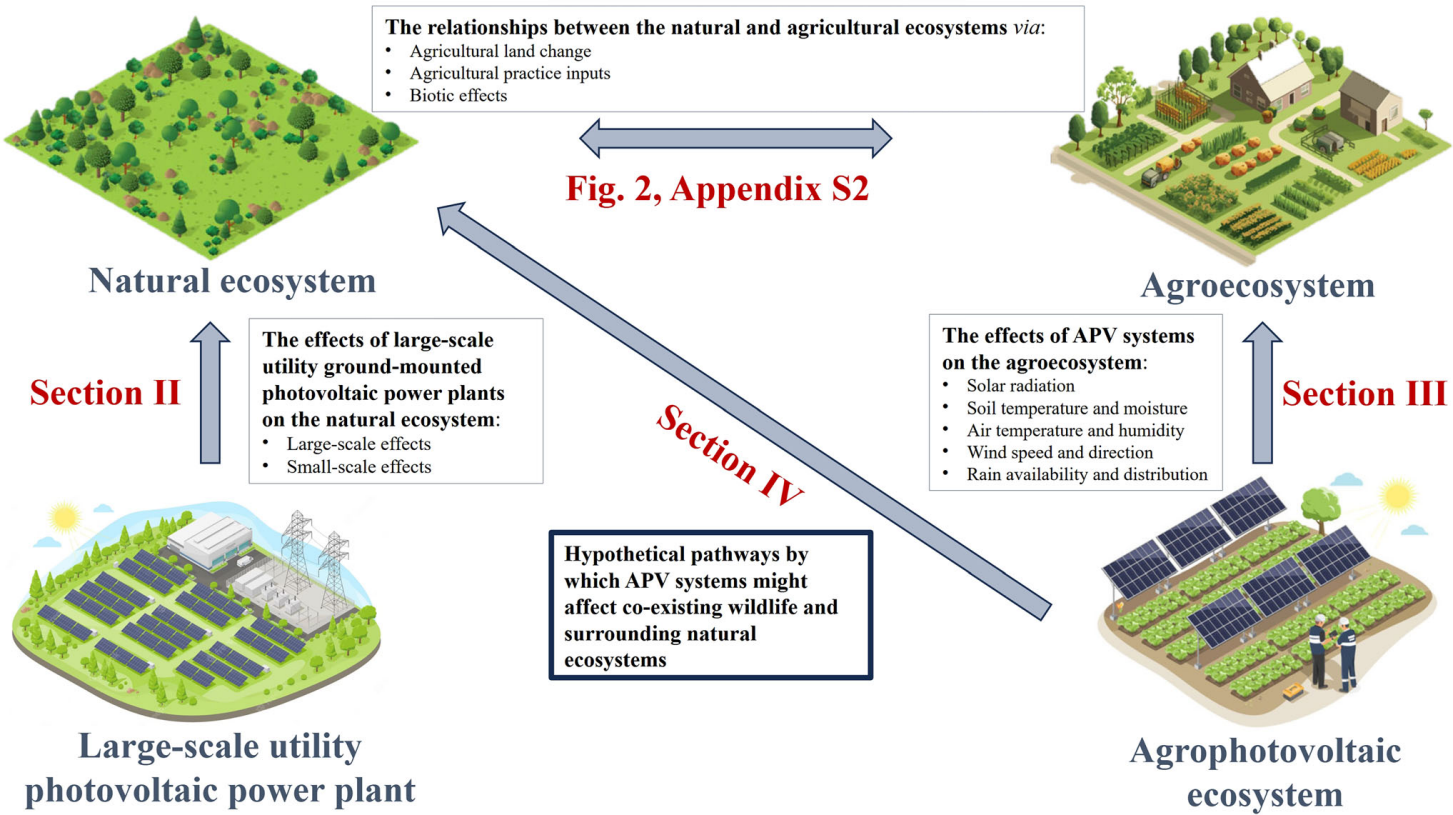
A scheme of the outline of this review. Figure 2 (and Appendix S2) explores the relationships between the natural and agricultural ecosystems *via* direct and indirect pathways. Section II reviews the existing knowledge about the large‐ and small‐scale effects of ground‐mounted utility‐scale photovoltaic (USPV) power plants on the natural ecosystem. Section III details the known microclimatic effects of agrovoltaic (APV) systems on the agroecosystem. Section IV synthesises this information and uses the established knowledge to hypothesise possible pathways by which APV systems might affect coexisting wildlife and natural ecosystems.

**Fig. 2:**
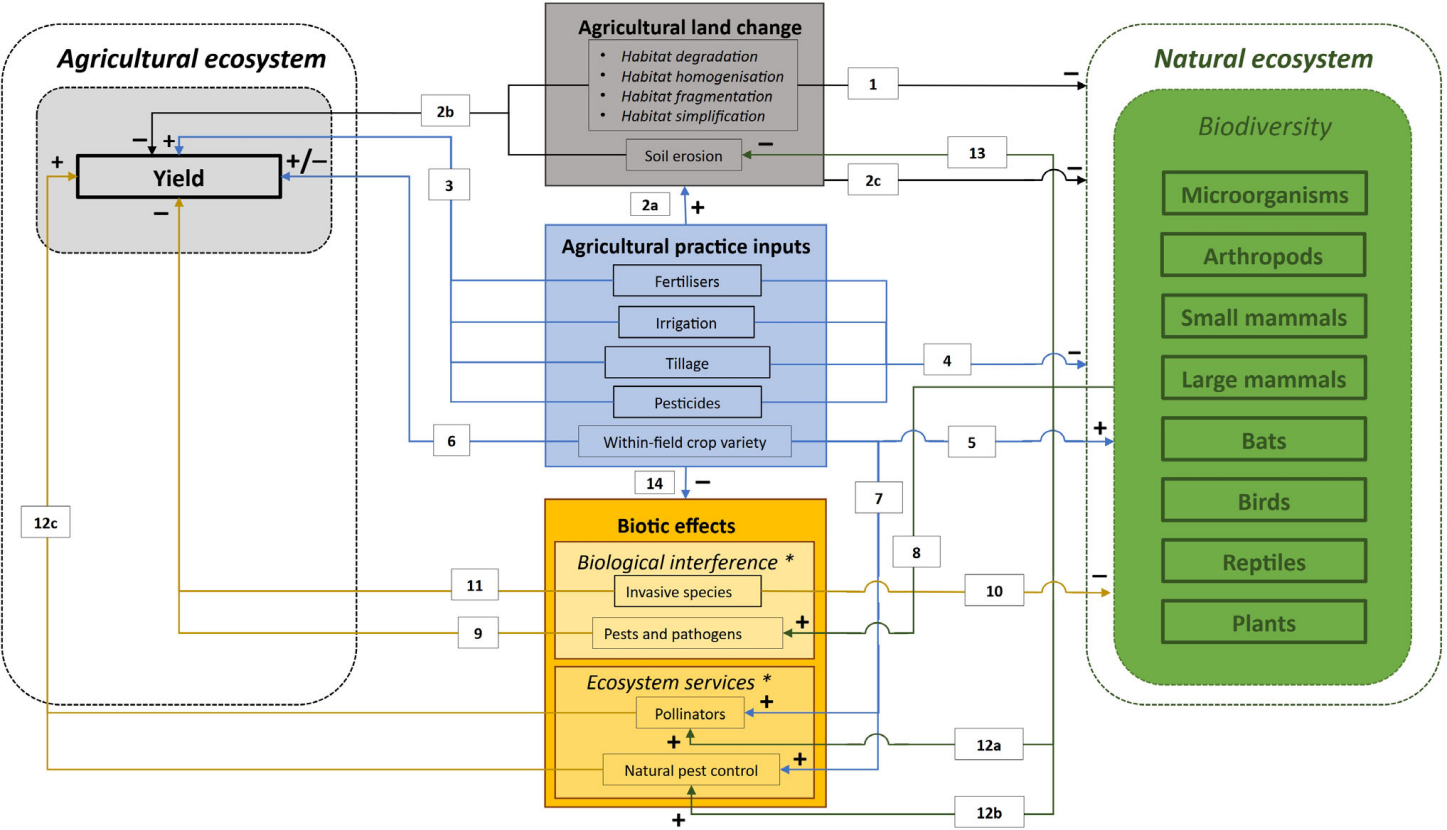
A scheme of the relationships between the natural and agricultural ecosystems. Factors related to agricultural land change, agricultural practice inputs, and biotic effects affect agricultural yield and biodiversity through direct and indirect pathways. The nature of the effect is indicated by + (positive effect), − (negative effect), and ± (mixed effects). Habitat degradation, homogenisation, fragmentation, and simplification follow the conversion of natural to agricultural habitat, and together with intensive agricultural practices, negatively impact the natural ecosystem (arrows 1 and 2a). This reduces biodiversity across spatial scales (2c), and in turn, negatively affects the agricultural yield (through soil erosion; 2b). Intensive agricultural practices and inputs, such as irrigation, tillage, and applying fertilisers, herbicides and pesticides, while beneficial to crop production in the short term (3), further negatively affect both coexisting wildlife and the neighbouring natural ecosystem (4). However, more wildlife‐friendly agricultural practices, such as increasing crop variety, can increase species and ecological diversity (5), promote pollinators and natural pest control (7), and thereby increase yield (6). Wild species can act as pests and pathogens (8), negatively affecting agricultural yield (9). Invasive or overabundant species can utilise agricultural lands to expand their range and penetrate novel natural ecosystems, potentially negatively impacting biodiversity through competition, predation or the spread of diseases or parasites (10), while simultaneously damaging agricultural yield (11). Other organisms, such as pollinators (12a) and natural enemies of pests (12b) provide ecosystem services, support agricultural production (12c), and prevent soil erosion (13). Intensive agricultural practice inputs, however, negatively affect wildlife species providing ecosystem services (14). For a more detailed explanation and references, see Appendix S2. * = Invasive species and pests and pathogens under biological interference, and pollinators and natural pest control under ecosystem services, are provided as examples of biotic effects. Many other possible biotic effects exist that we do not include for simplicity.

The environmental impact of APV may be studied in the context of Agroecology, an interdisciplinary science that combines ecological principles with agricultural practices to maximise both biodiversity and agricultural yield (Gliessman, 2019; see online Supporting Information, Appendix S1 for more details). The agroecosystem and natural ecosystem affect each other through complex pathways and interactions, mainly through land change effects, agricultural practices, and biotic interactions (Fig. 1). These well‐studied pathways and interactions are illustrated in Fig. 2 and discussed in more detail in Appendix S2.

In this review, we hypothesise novel potential pathways by which APV systems may impact wildlife and biodiversity. We aim to direct researchers towards studies and specific tests of possible effects of APV on biodiversity. We first provide a concise review on the impacts of ground‐mounted USPV power plants on the environment (Section II), and the effects of APV systems on the agroecosystem (Section III). We then hypothesise pathways by which PV panels on agricultural fields can impact biodiversity (Section IV). Finally, we conclude with recommendations for future research based on existing knowledge gaps (Section V).

## II. EFFECTS OF GROUND‐MOUNTED UTILITY‐SCALE PV (USPV) POWER PLANTS ON THE ENVIRONMENT

Electricity generation from solar energy *via* photovoltaic (PV) technology can impact the environment substantially in various ways (Abbasi & Abbasi, 2000; Tsoutsos *et al*., 2005; Chiabrando *et al*., 2009; IPCC, 2011; Lovich & Ennen, 2011; Turney & Fthenakis, 2011; Northrup & Wittemyer, 2013; Hernandez *et al*., 2014; Grippo, Hayse & O'Connor, 2015; Sánchez‐Zapata *et al*., 2016; Gasparatos *et al*., 2017; Guerin, 2017; Visser *et al*., 2019; Pimentel Da Silva, Magrini & Branco, 2020; Rabaia *et al*., 2021; Caprioli, Dell'Anna & Fiermonte, 2023). These impacts, however, are generally fewer and less severe than those associated with traditional power‐generation methods, such as use of fossil fuels (Dincer, 2000; Turney & Fthenakis, 2011).

Ground‐mounted USPV power plants typically have bare ground or short grass vegetation beneath the panels (e.g. turf grass; Walston *et al*., 2021). Such vegetation helps prevent overheating of the PV panels (see Section II.2.c below), and thus improves energy production efficiency, especially in semi‐arid and arid environments (Choi *et al*., 2023). This vegetation is often managed with pesticides or mowing to prevent shading of the panels and reduce fire risk (Uldrijan *et al*., 2021; Walston *et al*., 2021; Vaverková *et al*., 2022) and is usually less diverse compared to natural habitats (Uldrijan *et al*., 2021) unless sites intentionally attempt to enhance biodiversity (Walston *et al*., 2021; Blaydes *et al*., 2022; Tölgyesi *et al*., 2023). Reduced diversity is especially pronounced when power plants are established on previously natural habitats or recently abandoned agricultural lands, resulting in ecologically degraded sites (Hernandez *et al*., 2019; Uldrijan *et al*., 2021; Tölgyesi *et al*., 2023). However, power plants with turf grass can sometimes provide better ecological services than intensive mono‐cultural agricultural lands (Walston *et al*., 2021).

Some USPV power plants are experimenting with sowing and restoring species‐rich natural vegetation under the panels, a practice known as ecovoltaics, conservoltaics, or low‐impact solar (Montag, Parker & Clarkson, 2016; Semeraro *et al*., 2018; Sinha *et al*., 2018; Graham *et al*., 2021; Choi *et al*., 2023; McCall *et al*., 2023; Nordberg & Schwarzkopf, 2023; Sturchio & Knapp, 2023; Tölgyesi *et al*., 2023; Boscarino‐Gaetano, Vernes & Nordberg, 2024). This approach aims to mitigate negative microclimate effects, improve ecosystem services, and enhance biodiversity (Semeraro *et al*., 2018; Armstrong *et al*., 2021; Graham *et al*., 2021; Choi *et al*., 2023; Tölgyesi *et al*., 2023). Maintaining diverse vegetation can increase soil moisture, and reduce soil temperature and wind speeds, offering similar benefits to those observed in APV systems (see Section III; Sturchio *et al*., 2022; Choi *et al*., 2023). The focus on biodiversity in these managed areas has been shown to benefit pollinators (e.g. bumblebees and butterflies) and plant species diversity (Montag *et al*., 2016; Randle‐Boggis *et al*., 2020; Dolezal, Torres & O'Neal, 2021; Blaydes *et al*., 2022).

**Fig. 3:**
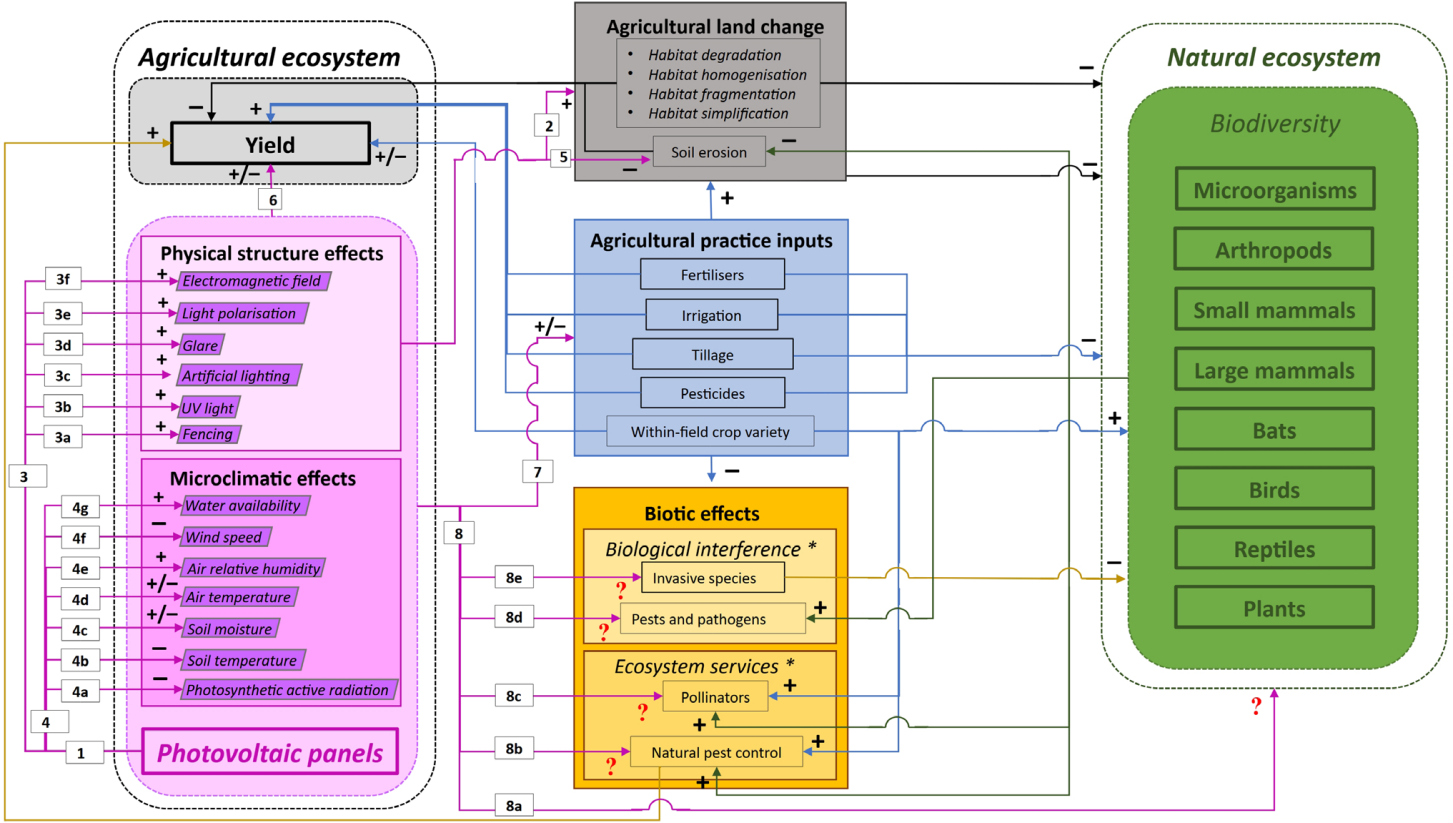
A scheme of the relationships between natural and agrophotovoltaic (APV) ecosystems. The addition of APV panels to an agricultural ecosystem introduces novel effects (pink shading, compare with Fig. 2), which can affect agricultural yield and biodiversity through direct or indirect pathways. The nature of the effect is indicated by + (positive effect), − (negative effect), and ± (mixed effects). Red question marks represent hypothetical pathways and effects that have not yet been studied. Note that pathways 1–3 are relevant for both utility‐scale photovoltaic (USPV) power plants and APV systems. See Sections II and III for discussion of the pathways indicated by the numbered arrows.

Ground‐mounted USPV power plants can have both large‐scale and small‐scale environmental effects (1 in Fig. 3).

### (1) Large‐scale effects of USPV power plants

Large‐scale effects include the overall impact of the USPV complex on the surrounding environment, such as the creation of heat islands, pollution, and disruption of habitat connectivity (Lovich & Ennen, 2011).

#### (a) Land conversion and destruction

Although solar energy production generally has a lower environmental impact compared to conventional or other renewable sources (Turney & Fthenakis, 2011; Mekhilef, Saidur & Kamalisarvestani, 2012), land use change during the installation phase is a primary environmental concern (2 in Fig. 3; Sánchez‐Zapata *et al*., 2016; Gasparatos *et al*., 2017; Pimentel Da Silva *et al*., 2020). Ground‐mounted PV power plants require land grading, compaction, and removal of topsoil (Pimentel Da Silva *et al*., 2020), which along with vehicular activity, can kill or entrap hibernating or aestivating animals (Lovich & Ennen, 2011). Vegetation is removed through pruning or herbicide application to minimise installation and operation costs, shading of the panels, and fire risk (Macknick, Beatty & Hill, 2013; Pimentel Da Silva *et al*., 2020). This leaves the soil degraded and susceptible to erosion (Hernandez *et al*., 2014). Allowing short vegetation to grow can reduce this erosion risk (Uldrijan *et al*., 2021; Walston *et al*., 2021).

Supporting infrastructure, such as access roads, electrical equipment, and grid connection, requires the destruction of additional land (Turney & Fthenakis, 2011). The degraded area of the power plant, its surrounding infrastructures, and related disturbances [e.g. dust, light pollution, microclimate changes, ultraviolet (UV) light] can negatively impact adjacent habitats (Turney & Fthenakis, 2011).

#### (b) Physical barriers

The transformation of open natural or agricultural habitats into PV power plants and their supporting infrastructure creates barriers for the movement of species, reducing connectivity between populations (3 in Fig. 3; Gasparatos *et al*., 2017), leading, for example, to loss of population genetic diversity (Saunders, Hobbs & Margules, 1991). Access roads and fencing, often necessary for security, further exacerbate habitat fragmentation by limiting wildlife movement through previously open ecological corridors (3a in Fig. 3; Chiabrando *et al*., 2009; Turney & Fthenakis, 2011; Caprioli *et al*., 2023). While some species may benefit from new hiding spots or perching sites (Fthenakis *et al*., 2011), or nesting sites within the power plant area (Hernandez *et al*., 2014), others, including opportunistic and non‐native species such as non‐native invasive species, may proliferate in the altered microclimate under the PV panels (Guerin, 2017; Pimentel Da Silva *et al*., 2020).

High‐voltage power lines connecting the plant to the grid can act as seemingly impenetrable barriers (Tyler *et al*., 2014), emitting UV light that can be detected by species including insects, birds, rodents, and reindeer (3b in Fig. 3; Hogg *et al*., 2011). This can cause avoidance behaviour (Tyler *et al*., 2014) and thus exacerbate habitat fragmentation (Vistnes *et al*., 2004).

#### (c) Pollution

The construction and operation of USPV power plants may involve the release of toxic chemicals, affecting the ground beneath the panels and surrounding terrestrial and aquatic areas through runoff (Grippo *et al*., 2015). Accidental releases of fuels, oils, and lubricants can occur during construction and maintenance (Lovich & Ennen, 2011). Pesticides and herbicides are commonly used to control vegetation, potentially impacting nearby ecosystems (Lovich & Ennen, 2011; Guerin, 2017). Dust accumulation on PV panels reduces their performance (Lovich & Ennen, 2011), thus necessitating the usage of dust suppressants (Abbasi & Abbasi, 2000; Lovich & Ennen, 2011), which may contain harmful chemicals (e.g. brines, salts) and contribute to pollution in adjacent habitats, including aquatic ecosystems (White & Broadley, 2001; Singh, Piechota & James, 2003; Grippo *et al*., 2015).

### (2) Small‐scale effects of USPV power plants

Small‐scale effects include impacts on the immediate microenvironment under the PV panels and its supporting structures and coexisting and incidental wildlife (Lovich & Ennen, 2011).

#### (a) Light pollution

Light pollution from USPV power plants can include artificial light at night, glare, and polarised light (Chiabrando *et al*., 2009; Lovich & Ennen, 2011), all of which can affect the biology and ecology of wildlife (Longcore & Rich, 2004). Artificial light can disrupt nocturnal wildlife and interfere with navigation, behaviour and physiological processes (3c in Fig. 3; Longcore & Rich, 2004). Glare from sunlight reflecting off PV panels can temporarily impair vision in both humans and animals (3d in Fig. 3; Chiabrando *et al*., 2009).

Polarised light, which appears similar to reflections from water surfaces, can attract organisms such as birds, reptiles, amphibians, and insects to the artificial surface, where they may also oviposit (3e in Fig. 3; Wehner & Labhart, 2006; Horváth *et al*., 2009). Thus PV panels can act as ecological traps (Schlaepfer, Runge & Sherman, 2002), resulting in reproductive failures (Horváth *et al*., 2009, 2010) and mortality through collision (Kagan *et al*., 2014) or predation by other species (Kagan *et al*., 2014; Walston *et al*., 2016; Visser *et al*., 2019).

#### (b) Electromagnetic field effects

Electromagnetic fields generated by PV plant infrastructure (3f in Fig. 3; Chiabrando *et al*., 2009; Balmori, 2010), also may affect wildlife. Potential impacts include disruption of the nervous system, circadian rhythms, heart functions, immune response, and fertility in wild mammals (Balmori, 2010), as well as altered pollination behaviour and efficiency in honeybees (Molina‐Montenegro *et al*., 2023). However, these effects remain debated, and require further research (Lovich & Ennen, 2011).

#### (c) Altered microclimate

The construction of PV power plants involves clearing vegetation, altering the local microclimate by reducing evapotranspiration and causing elevated temperatures beneath the panels, leading to a photovoltaic heat island effect (Barron‐Gafford *et al*., 2016). This effect can decrease solar panel efficiency (Skoplaki, Boudouvis & Palyvos, 2008), and deter some species (Pociask & Fuhr, 2011), while potentially attracting others, including invasive species (Battles & Kolbe, 2019). Conversely, in arid environments, the shade from PV panels can create a cool island effect that may extend to the outer perimeter of the power plant, potentially providing a thermal refuge (Chang *et al*., 2018; Guoqing *et al*., 2021). Both types of effects may have positive or negative consequences for biodiversity and ecosystem processes.

## III. EFFECTS OF APV SYSTEMS ON THE AGROECOSYSTEM

Despite a growing body of literature on the effects of ground‐mounted USPV power plants on biodiversity and ecosystem services (e.g. Armstrong *et al*., 2014, 2016; de Marco *et al*., 2014; Randle‐Boggis *et al*., 2020; Lafitte *et al*., 2023), there is still very little understanding of how different aspects of USPV and APV systems affect biodiversity (Uldrijan *et al*., 2021). According to a recent review (Lafitte *et al*., 2023), most studies have considered impacts on species abundance, composition, and diversity of plants and arthropods in temperate climates. Studies investigating the effects of PV systems on vertebrates, and on physiology and behaviour, are still scarce (Lafitte *et al*., 2023). Much more knowledge is available on how APV systems affect agroecosystems, including cultivated crops. This allows us to hypothesise potential impacts of APV systems on co‐occurring wildlife species.

### (1) Microclimate alterations under APV systems

APV systems, which place PV panels above agricultural lands, may alter the microclimate differently from panels installed over bare ground (4a–4g in Fig. 3; Barron‐Gafford *et al*., 2016, 2019). Key differences include impacts on photosynthetically active radiation (PAR), air and soil temperatures, relative humidity, soil moisture, wind speed, and water availability (Al Mamun *et al*., 2022). The impacts on these will depend on the type of PV panels, their size, tilt, installation height, and the spacing between arrays (Beck *et al*., 2012; Marrou *et al*., 2013b; Weselek *et al*., 2021).

#### (a) Photosynthetically active radiation (PAR)

PAR under PV panels in APV systems is significantly reduced, often 30–75% lower, compared to unshaded areas, particularly during the morning and in the summer (4a in Fig. 3; Patel & Chauhan, 2009; Beck *et al*., 2012; Marrou *et al*., 2013b; Adeh, Selker & Higgins, 2018; Weselek *et al*., 2021; Al Mamun *et al*., 2022). The extent of this reduction is influenced by various factors, including the time of day, latitude, season, module type, tilt angle, installation height and spacing between arrays (Marrou *et al*., 2013b; Elborg, 2017; Santra, Meena & Yadav, 2021; Trommsdorff *et al*., 2021). The reduced solar radiation under PV panels is a major limitation for crop production (Beck *et al*., 2012; Marrou *et al*., 2013b; Santra *et al*., 2017; Gonocruz *et al*., 2021; Weselek *et al*., 2021). In APV systems where panels are elevated on stilts, more sunlight reaches the ground, benefiting plant growth compared to ground‐mounted APV systems (Marrou *et al*., 2013c; Elborg, 2017; Kumpanalaisatit *et al*., 2022).

#### (b) Soil temperature and moisture

Soil temperature under APV systems is typically lower due to shading (4b in Fig. 3; Marrou *et al*., 2013b; Weselek *et al*., 2021), which can be beneficial during hot periods but may delay crop maturation in cooler seasons (Weselek *et al*., 2021). Soil moisture in APV systems tends to be lower than in open fields in temperate climates during the winter (4c in Fig. 3; Weselek *et al*., 2021), but higher in semi‐arid regions, where APV systems enhance water‐use efficiency (Adeh *et al*., 2018; Jain *et al*., 2021).

#### (c) Air temperature and relative humidity

Air temperatures are generally lower under APV panels compared to bare‐ground‐mounted PV panels (Patel & Chauhan, 2009; Adeh *et al*., 2018; Weselek *et al*., 2021), where a photovoltaic heat island effect can occur (Barron‐Gafford *et al*., 2016). In some cases, depending on solar radiation and wind speed, air temperatures below APV modules were not different from open‐field air temperatures (4d in Fig. 3; Marrou *et al*., 2013b). Relative humidity tends to be higher under the panels of APV systems than in open fields (Weselek *et al*., 2021), especially during winter, which can influence plant growth and soil processes (4e in Fig. 3; Patel & Chauhan, 2009).

#### (d) Wind speed and water availability

APV structures reduce wind speed and serve as windbreaks (4f in Fig. 3; Adeh *et al*., 2018; Barron‐Gafford *et al*., 2019; Weselek *et al*., 2021; Choi *et al*., 2023), potentially reducing soil erosion (5 in Fig. 3; Jain *et al*., 2021). Water availability from rain is uneven below APV structures, and varies according to panel movement, tilt angle, and spacing between arrays (4 g in Fig. 3; Elamri *et al*., 2018; Weselek *et al*., 2021). Some APV arrays have rainwater‐harvesting systems that collect water from the panels, cleaning them from dust, and redistributing it for irrigation of the crops below (Santra *et al*., 2018; Jain *et al*., 2021). This optimises water usage, and removes the need to use pollutant chemicals for dust removal (Santra *et al*., 2018; Jain *et al*., 2021).

### (2) Agricultural implications

APV systems can result in delayed crop development, for example in wheat (Marrou *et al*., 2013b; Weselek *et al*., 2021), where growth under shade leads to taller, slower‐growing plants (Li *et al*., 2010; Weselek *et al*., 2021). Their impact on agricultural yield varies, with potential reductions in cooler seasons, especially in temperate climates (6 in Fig. 3; Oleskewicz, 2020; Weselek *et al*., 2021) or for shade‐intolerant crops (Jo *et al*., 2022; Lee *et al*., 2022). Conversely, yield increases have been reported in hot, dry climates (e.g. in the summer months or desert climates), and windy sites, or for shade‐tolerant crops (Adeh *et al*., 2018; Barron‐Gafford *et al*., 2019; Weselek *et al*., 2021). The selection of crops and the configuration of PV panels (e.g. angle, height, and orientation) are crucial in optimising both agricultural and electrical yields (Beck *et al*., 2012; Marrou *et al*., 2013, 2013; Elborg, 2017; Santra *et al*., 2017; Valle *et al*., 2017; Sekiyama & Nagashima, 2019; Cho *et al*., 2020; Schindele *et al*., 2020; Zheng *et al*., 2021; Jo *et al*., 2022; Katsikogiannis, Ziar & Isabella, 2022; Kumpanalaisatit *et al*., 2022).

The unique microclimates under APV systems require adjustments to agricultural practices (7 in Fig. 3). A preference for shade‐tolerant crops could influence crop rotation and variety selection, potentially affecting the use of pesticides, fertilisers, and tillage practices. However, due to the relatively new adoption of APV systems, comprehensive guidelines for these adjustments are still in development.

In summary, while APV systems offer potential benefits for renewable energy and agriculture, their impacts on biodiversity, microclimate, and agricultural practices require further investigation. Understanding these effects will help optimise APV system design and management for sustainable agriculture and biodiversity conservation.

## IV. KNOWLEDGE GAPS AND HYPOTHESISED PATHWAYS BY WHICH APVS MIGHT AFFECT BIODIVERSITY

Based on the known effects of ground‐mounted PV panels on the environment and those of APV systems on agricultural production, as detailed in Sections II and III, we now hypothesise potential impacts of APV systems on biodiversity. These hypothesised effects and pathways (Table 1), include the influence of the APV system's physical structure, pollution (e.g. light, electromagnetic), and altered microclimate. The nature and direction of these effects (positive or negative) will likely depend on the ecological context and the specific traits of the species involved. These proposed pathways provide a foundation for future research.

**Table 1:** Summary of the proposed effects and pathways by which agrophotovoltaic (APV) systems are hypothesised to affect biodiversity. The table details the potential effects of APV systems based on existing literature on the impacts of utility‐scale PV (USPV) power plants on biodiversity (Section II), the effects of APV systems on agricultural crops (Section III), and the hypothesised effect (positive/negative/mixed) these may have on biodiversity (Section IV).

Altered variable	APV feature causing impact	Taxa impacted	Impact	Likely route of impact	Outcome	Potential effect on biodiversity
Land change, habitat conversion and destruction	Installing APV systems on existing agricultural fields	All	Positive	Prevention of further natural habitat conversion Providing shade, shelter, and water access	Increased shelter availability Refugia for rare species	Positive
			Negative	Agroecological microhabitat conversion	Changed species composition, diversity and abundance Increased local extinction	Negative
Physical structure	APV panels, stilts, fences, supporting structures, added artificial habitat structures	Prey species	Positive	Protection from predators	Increased shelter and nesting site availability	Positive
		Non‐native species/local overabundant species	Positive	Creation of favourable microclimatic conditions Protection from predators	Establishment Range expansion Becoming agricultural pests Increased predation on local wildlife Increased competition with local wildlife	Negative
		Flying species	Negative	Collision with structures	Reduced natural pest control	Negative
		Predator species	Positive	Creation of perching sites	Increased predation pressure	Negative
					Increased natural pest control	Positive/negative
	Supporting structures (power lines emitting UV light)	Insects, birds, rodents, and large mammals	Negative	Avoidance of APV systems Avoidance of power line perimeter	Reduced natural pest control Reduced movement ability Reduced habitat connectivity	Negative
	Fences	Large flightless species	Negative	Reduced habitat connectivity	Disrupted migratory routes Reduced genetic diversity	Negative
Pollution	Artificial light at night	Nocturnal species	Negative	Disruption of circadian rhythm	Behavioural changes Species composition changes	Negative
		Non‐native species/local overabundant species	Positive	Increased food availability Penetration of novel environments	Increased predation on local wildlife Increased competition with local wildlife	Negative
	Glare	Birds	Negative	Disruption of navigation abilities	Interruption of migration flyways	Negative
	Polarised light	Birds, reptiles, amphibians, and insects	Negative	Attraction to PV panel (mistaken as water body)	Collision with structures Reproductive effort wasted Increased predation	Negative
	Electromagnetic field generated by grid cables	Wild mammals	Negative	Physiological effects	Damage to the nervous system Disruption to circadian rhythms Changes to heart functions Impaired immunity and fertility Genetic and developmental problems	Negative
	Fuels, oils, lubricants, dust suppressants				Changed species composition, diversity and abundance Local extinction Runoff to adjacent habitats	
Microclimate changes	Arid and mesic environments	Shade‐tolerant plant, fungal and microorganism species	Positive	Increased soil moisture Reduced photosynthetically active radiation Reduced soil temperature Reduced air temperature Increased relative humidity	Altered growth rates and patterns Later or earlier seasonal blooming Fewer or more annual seasonal blooming events Modified nutrient content Refugia for rare species Mitigation of climate change effects Increased competition with crops Increase in natural enemies and pathogens	Positive
					Increased use of pesticides and herbicides	Negative
		Shade‐intolerant plant, fungal and microorganism species	Negative		Changed species composition Exclusion by competition Local extinction	Negative
		Animal species	Positive/negative		Changed behaviour Changed phenology Changes in life‐history traits	Positive/negative
		Pollinators	Positive/negative		Altered activity patterns Changed foraging behaviour Altered pollination efficiency	Positive/negative
		Non‐native species/local overabundant species	Positive		Becoming established Expanding ranges Becoming agricultural pests Increased predation on local wildlife Increased competition with local wildlife	Negative

The physical structures and the microclimate created by APV systems can directly affect the local fauna and flora (8a–e in Fig. 3). These impacts may be direct, affecting wildlife present within the APV field (8a in Fig. 3), or indirect *via* effects on species that provide natural pest control (8b in Fig. 3), act as pollinators (8c in Fig. 3), or are pests or pathogens (8d in Fig. 3). The presence of invasive species (8e in Fig. 3) may also be directly or indirectly affected. The effects of APV systems will depend on the existing biodiversity, local climate, and agricultural practices (Hernandez *et al*., 2014). For some species the APV environment may be more attractive, while others may avoid it, potentially causing cascading effects on surrounding habitats and affecting biodiversity on a large scale (8a in Fig. 3).

### (1) Land change

The primary environmental concern regarding the construction of new ground‐mounted PV power plants is habitat destruction, especially when they are sited in previously natural areas (Sánchez‐Zapata *et al*., 2016; Gasparatos *et al*., 2017; Pimentel Da Silva *et al*., 2020). By contrast, APV systems, which elevate PV panels above agricultural fields using stilts, by placing them between crops, or designing the solar farm to include retained or restored vegetated patches within its boundaries (Boscarino‐Gaetano *et al*., 2024), require minimal additional land disruption. Thus, APV systems installed on stilts may affect local biodiversity less than ground‐mounted systems. However, the introduction of APV structures to the agroecological habitat may still alter species diversity, abundance and composition, potentially causing local extinctions. The extent to which stilt‐mounted APV systems are less disruptive to the surrounding ecosystems than ground‐mounted APV or USPV power plants over various spatial and temporal scales remains to be investigated.

### (2) Physical structures and barrier effects

Many of the impacts observed in ground‐mounted USPV power plants (see Sections II.1.a and II.1.b) are likely to be present also in APV systems. PV panels, stilts, and related infrastructure (e.g. grid cables, fences) increase the structural complexity and heterogeneity of the agricultural ecosystem (Nordberg *et al*., 2021; Uldrijan, Winkler & Vaverková, 2023). These structures, as well as artificial habitat structures that may be added (e.g. nest boxes, burrows, hollows and hibernacula), could potentially benefit some prey species by providing shelter and protected nesting sites, or by acting as a barrier to predators (Table 1; Fthenakis *et al*., 2011; Hernandez *et al*., 2014; Sinha *et al*., 2018; Nordberg *et al*., 2021; Boscarino‐Gaetano *et al*., 2024). However, they could also attract invasive or overabundant species, which might exploit the disturbed area (Guerin, 2017; Pimentel Da Silva *et al*., 2020), potentially becoming pests and displacing or predating local wildlife (Table 1).

Conversely, APV structures might serve as perching sites for predators, increasing predation pressure, but also providing natural pest control (DeVault *et al*., 2014; Sinha *et al*., 2018; Nordberg *et al*., 2021). This could lead to APV systems becoming ecological traps for both prey and predator species (e.g. Rotem *et al*., 2013), attracting them into a hazardous environment, where they are at a higher risk of predation or injury from collision with APV structures (Table 1; Visser *et al*., 2019). Predators that forage in open fields and avoid structurally complex habitats, may be excluded from the APV area (Table 1), subsequently reducing their provision of ecosystem services, such as natural pest control (Table 1). Fencing around APV fields could disrupt the movement of larger animals, affecting habitat connectivity and disrupting migratory routes (Gasparatos *et al*., 2017). This could lead to large‐scale effects on populations, and even result in local extinctions (see Section II.1.b; Saunders *et al*., 1991; Chiabrando *et al*., 2009; Turney & Fthenakis, 2011; Caprioli *et al*., 2023). It remains uncertain how these dynamics will play out in the agricultural context of APV systems, and future detailed studies are needed.

### (3) Pollution

APV systems may include artificial lighting for maintenance, creating light pollution in a previously rural habitat, disrupting nocturnal wildlife, and potentially attracting invasive and overabundant species (see Section II.2.a). Thus, community assemblages may be affected. The glare from PV panels can also interfere with bird navigation, disrupting migration flyways (Table 1). Polarised light from the panels may attract some species, leading to collisions, predation, and wasted reproductive effort (see Section II.2.a; Horváth *et al*., 2009).

Chemical pollution risks are potentially lower in APV than in USPV systems, due to the presence of vegetation reducing dust, and thus the need for dust‐repellent chemicals (see Section II.1.c; Lovich & Ennen, 2011). Ground pollution in an APV system is undesirable for the farmer because it may harm agricultural products, and chemicals that are hazardous for consumption are less likely to be used. However, any spills during construction or maintenance could still impact wildlife (see Section II.1.c; Lovich & Ennen, 2011). Comparisons of the pollution risks between APV and USPV systems are required to determine their significance for the surrounding ecosystems.

### (4) Microclimate effects

The altered microclimate beneath PV panels in APV systems may affect relatively sessile species (e.g. plants, fungi, microorganisms) in a similar way to effects on crops. Reduced PAR, lower soil and air temperatures, and increased soil moisture and relative humidity under the panels may favour shade‐tolerant species, especially in arid regions (see Section III.1; Table 1). This altered environment could create refugia for rare species, potentially mitigating some impacts of climate change (Rosenzweig, 2003), and assist with soil restoration (Heredia‐Velásquez *et al*., 2023). Conversely, species requiring more sunlight may be excluded from these shaded areas. The heterogeneous microclimate within APV fields could create differences in species assemblages and abundances compared to open fields. Additionally, altered microclimates under APV panels can modify growth rates and patterns, phenology, and nutrient content of wild plants (Graham *et al*., 2021), potentially influencing the behaviour, phenology, and life history of herbivores and pollinators (Graham *et al*., 2021). Some wild plants may compete with crops for sunlight, while fungal species that thrive in shaded and moist conditions may benefit, reducing crop yield. These effects may necessitate the increased use of herbicides or pesticides, with consequent cost implications. The increased localised water availability and shade under PV panels in APV fields may also create favourable conditions for invasive or overabundant species, potentially exacerbating competition with native species, reducing biodiversity. They may potentially also become agricultural pests.

The extent to which wildlife in APV systems will mirror the responses of crops to these microclimatic changes remains an open question. Additionally, it is uncertain how agricultural practices, including pest and vegetation management, will interact with these dynamics. Further research is needed to understand these interactions and to develop guidelines for managing biodiversity in APV systems.

## V. FUTURE RESEARCH DIRECTIONS

In Section IV we proposed new hypotheses and potential pathways by which APVs might influence biodiversity, identifying significant knowledge gaps. There is currently little evidence available on the impacts of APVs on biodiversity, particularly empirical studies. Given the rapid global expansion of APVs technology, there is an urgent need to expand our understanding and establish clear research pathways to ensure biodiversity conservation alongside these emerging structures. The hypothesised effects of APVs on biodiversity, as well as the mechanisms and pathways by which these effects may operate, are likely to vary significantly depending on factors such as geographic location, climate, local biodiversity and agricultural practices. This complexity presents a plethora of research opportunities, which we outline here. Key questions are summarised in Table 2, and detailed further below.

**Table 2:** Potential research questions for future studies, focusing on the effects of agrophotovoltaic (APV) systems on small and large scales, and on their technical and agricultural aspects.

Small‐scale effects
(1) How do changes in physical and/or biological conditions imposed by APV structures affect biodiversity? (2) What mechanisms and relationships are promoted or depressed in APV systems compared to agroecological ecosystems? (3) Do species interactions change in APV fields and surrounding environments? (4) What are the effects of APV systems on functional groups (e.g. predators, pollinators, decomposers, pathogens) and their interactions?
Large‐scale effects
(5) How do any effects on biodiversity change in APV systems across different geographic zones, climates and habitat types? (6) What are the complex, large‐scale spatial effects of APV structures on macroecological processes (e.g. species distributions, dispersal, migration patterns)? (7) How is global warming predicted to impact the effects of APV systems on biodiversity and on vulnerable species across different habitat types? (8) Is ecovoltaics superior to APVs in promoting biodiversity, ecosystem functioning and ecosystem services across habitats?
Technical and agricultural aspects of APV systems
(9) Do any crops grown in APV systems benefit biodiversity and surrounding ecosystems more than others? (10) How do the supporting structures of APV systems (e.g. fencing, grid cables, stilts) impact biodiversity? (11) Do different PV panel technologies (e.g. semi‐transparent panels, sun‐tracking technologies) affect biodiversity differently? (12) What wildlife‐friendly practices minimise negative impacts and enhance positive impacts of APVs on biodiversity, and on vulnerable taxa? (13) How can research‐based planning of APV systems (e.g. geographic location, system layout, surveys of the types of species and habitats present, addition of artificial habitat structures) enhance biodiversity?

Future research should encompass both small‐scale empirical studies and large‐scale macro‐ecological studies, including meta‐analyses. A crucial approach would be to establish long‐term research plots within and adjacent to APV fields. Such approaches would provide essential data on the abiotic and biotic impacts of APV systems, capturing both transitional and steady‐state patterns over time.

The integration of APV systems in agricultural fields presents an opportunity simultaneously to support biodiversity and agricultural production. The effects on biodiversity are likely to be influenced by the specific methods of installation, the types of technology employed, and the agricultural practices utilised, all of which could lead to either positive or negative outcomes for biodiversity. Therefore, it is crucial to conduct comprehensive and nuanced research to understand fully the direct and indirect effects of APV systems, enabling informed decisions that balance ecological and agricultural benefits.

### (1) Baseline assessments and long‐term monitoring

Future research should include a baseline assessment of biodiversity before and during installation of the APV system, and continue with long‐term monitoring (Q1–Q4 in Table 2). This approach will enable both immediate and gradual changes in the abiotic and biotic environments due to APV systems to be assessed. Studies also should compare biodiversity in APV plots with adjacent reference plots to determine whether observed changes are temporary or indicate more permanent shifts in ecosystem structure and function.

### (2) Spatial and temporal scales of biodiversity impact

Future research must investigate whether APV impacts differ across different regions and scales. This includes understanding how local impacts vary with regional factors such as climate, habitat type, and agricultural practices, as well as different APV technologies. These studies should focus on the effects on functional groups such as predators, pollinators, decomposers, and pathogens, and assess overall biodiversity impacts (Table 2). Additionally, large‐scale research can provide insights into broader ecological processes, such as species distributions, movement ecology, and migration patterns affected by APV structures (Table 2, Q5 and Q6).

### (3) APVs and biodiversity under global change

Future studies should explore how APV systems might benefit or harm species, particularly those that are endangered or vulnerable, in the context of global change (Table 2, Q7). Research should consider how climate change might interact with APV systems to influence biodiversity outcomes. Understanding these dynamics is crucial for predicting and managing the impacts of APV systems on biodiversity across diverse habitats.

### (4) Comparative analysis of APV and ecovoltaic systems

A comparative research approach should be employed to study biodiversity metrics, ecosystem functions and services provided by APV systems *versus* ecovoltaic systems, that is PV systems that purposefully enhance biodiversity (Table 2, Q8). Such comparisons will be critical for understanding the potential benefits or drawbacks of each system in various environmental and socio‐economic contexts, and to guide decisions on suitability for specific contexts.

### (5) Technical and agricultural aspects of APV systems

Future research should examine the impact of different crops, such as perennials *versus* annuals, on biodiversity within APV systems and their surrounding environments (Table 2, Q9). The influence of different technical attributes of APV panels (e.g. semi‐transparent panels; Gorjian *et al*., 2022), their supporting structures (e.g. sun‐tracking technologies *versus* fixed systems; Valle *et al*., 2017), design (e.g. ground‐mounted *versus* stilt‐mounted) and infrastructure (e.g. fences, grid cables) on local flora and fauna should be explored (Table 2, Q10 and Q11). Such studies could provide information on the technical requirements and limitations of different APV technologies and designs. These impacts can then be compared between geographic and climate zones, and be integrated with research on the economic profitability of different crops, technologies and practices, and their social impacts.

### (6) Enhancing biodiversity through wildlife‐friendly practices and research‐based planning

Identifying and promoting wildlife‐friendly agricultural practices within APV systems, as well as planning the APV system to include natural patches or artificial habitat structures, will be vital for minimising negative effects and enhancing positive impacts on biodiversity. Some practices, such as reduced pesticide use or limited tillage, and the implementation of research‐based planning of APV systems, may be particularly beneficial for vulnerable species and overall ecosystem health. Identifying and implementing these practices could significantly improve the sustainability of APV systems, supporting both agricultural productivity and biodiversity conservation (Table 2, Q12 and Q13).

## VI. CONCLUSIONS

(1) APV systems offer a dual‐use solution that offers an alternative to the conversion of open habitats and existing agricultural fields into traditional PV power plants, providing a sustainable approach to land use.
(2) There is a significant need for detailed studies to explore the largely unknown impacts of APV systems on biodiversity, particularly in comparison to other photovoltaic systems.
(3) With the increasing implementation of APV technology worldwide, extensive research is essential to understand the complex interactions between APV systems, agroecosystems, and natural ecosystems.
(4) Future research should consider various potentially interacting factors, including habitat type, climate, spatial scale, technology, agricultural practices and the effects of climate change, to assess accurately the biodiversity impacts of APV systems.
(5) A wide range of research areas, from initial assessments and long‐term monitoring to the study of technological and agricultural influences on biodiversity, must be explored to determine the overall effects of APV systems on ecological health.

## AUTHOR CONTRIBUTIONS

R. S.: conceptualisation, writing of original draft, figure drawing, review & editing; Y.Z.: supervision, funding, conceptualisation, writing of original draft, review & editing.

## Supporting information


**Appendix S1.** Agroecology definition.

**Appendix S2.** Agroecosystem: the relationships between natural and agricultural ecosystems.
