# Supplementary material for: Shedding light on biodiversity: reviewing existing knowledge and exploring hypothesised impacts of agrophotovoltaics
Source: Biol Rev Camb Philos Soc. 2024 Nov 10;100(2):855–70. doi: 10.1111/brv.13165 (PMC11885692; doi:10.1111/brv.13165)
Supplement: Supplementary file 1 — Appendix S1. Agroecology definition. [file BRV-100-855-s001.docx]

**Appendix S1. Agroecology definition**

Agroecology is a multidisciplinary field of study that combines principles of ecology, agronomy, and social sciences to design and manage agricultural systems that are environmentally friendly, economically viable, and socially just (Wezel *et al*., 2009; Gliessman, 2018). The term ‘agroecology’ was originally used to describe the ecological study of agricultural systems and their relationship with the broader environment (Gliessman, 2007; Wezel & Soldat, 2009). More recently, this science has evolved to include the adoption of wildlife-friendly agricultural practices and the social and economic dimensions of food systems (Francis *et al*., 2003). Agroecology aims to minimise the negative impacts of agriculture on the environment, while promoting biodiversity, soil health, and ecosystem resilience (Gliessman, 2019) with minimal compromise with food production. It emphasises the integration of ecological processes, such as nutrient cycling, natural pest control, and crop diversification, into farming practices (Gliessman, 2019). It also focuses on empowering local communities, promoting equitable access to resources, and considering agriculture’s social and cultural dimensions (Kerr *et al*., 2021). Agroecology is seen as an alternative to conventional industrial and intensified agriculture, which relies heavily on synthetic inputs, monocultures, and mechanisation (Chappell & LaValle, 2011; Gliessman, 2019). Proponents argue that agroecological approaches that enhance ecosystem services can lead to greater food security, biodiversity conservation, climate change mitigation, and social well-being (Gliessman, 2018; Amoak, Luginaah & McBean, 2022).
