# Supplementary material for: Shedding light on biodiversity: reviewing existing knowledge and exploring hypothesised impacts of agrophotovoltaics
Source: Biol Rev Camb Philos Soc. 2024 Nov 10;100(2):855–70. doi: 10.1111/brv.13165 (PMC11885692; doi:10.1111/brv.13165)
Supplement: Supplementary file 2 — Appendix S2. Agroecosystem: the relationships between natural and agricultural ecosystems. [file BRV-100-855-s002.docx]

**Appendix S2. Agroecosystem: the relationships between natural and agricultural ecosystems**

Fig. 2 presents major pathways and interactions, numbered as detailed herein. The agricultural and natural ecosystems are connected *via* three major effects: agricultural land change (detailing large- and small-scale land-conversion implications), agricultural practice inputs (including the major inputs and practices farmers apply in agricultural fields), and biotic effects (including both negative influences and beneficial ones). It is important to emphasise that any conceptual model cannot capture the full complexity of ecological interactions and existing factors. We include here the most important pathways and interactions, but note that many other potential interactions, pathways and underlying processes exist.

The conversion of natural habitat to agricultural land is followed by habitat degradation, homogenisation, fragmentation, and simplification, which negatively impact the natural ecosystem and reduce biodiversity across spatial scales (arrow 1 in Fig. 2; Tscharntke *et al*., 2005, 2012; Bommarco, Kleijn & Potts, 2013). In the process of habitat conversion, the natural vegetation is removed, the ground is levelled, the topsoil is modified and left bare and more sensitive to erosion. Ensuing intensive agricultural practices intended to maximise yields, such as tillage, short crop rotations of monoculture, and extensive irrigation, further exacerbate soil erosion processes (2a in Fig. 2). Soil erosion negatively impacts both agricultural productivity (2b in Fig. 2) and wildlife (2c in Fig. 2; Gliessman, 2019). Intensive agricultural practices inputs, such as irrigation, tillage, and applying fertilisers and pesticides, while beneficial to crop production in the short term (3 in Fig. 2), further negatively affect both coexisting wildlife and the neighbouring natural ecosystems (4 in Fig. 2; Chiron *et al*., 2014). Tillage and the application of pesticides and herbicides reduce or prevent the co-occurrence of wild species of plants and animals in agricultural fields, leading to biodiversity loss and species composition changes (Benton, Vickery & Watson, 2003; Sharma, Jha & Reddy, 2018). When fertilisers spill into natural habitats, they can encourage the overgrowth of species that can take temporary or permanent advantage of the elevated nutrient levels to become dominant at the expense of species biodiversity (Tamm, 1991). Such population increases, e.g. algal blooms in natural water systems, can result in the release of harmful toxins (Hallegraeff, 2003). However, more wildlife-friendly agricultural practices, such as increasing crop variety, can increase the heterogeneity of the agroecosystem, thus providing more diverse microhabitats, niches, and food for wildlife, thereby increasing species and ecological diversity (5 in Fig. 2; Gliessman, 2019). Increasing crop variety reduces the yield of each specific crop grown concurrently and may make it impossible for farmers to use certain machinery, thus increasing labour costs (6 in Fig. 2; Power & Follett, 1987). But it may also indirectly increase total agricultural yield in the long term by increasing the abundance of pollinator species and natural enemies or competitors of pests and pathogens, and by prevention of soil erosion (7 in Fig. 2; Chaplin-Kramer *et al*., 2011).

The natural and agricultural agroecosystems also interact *via* biotic factors, with both adverse and beneficial outcomes to agricultural yield and biodiversity (Jarvis, Padoch & Cooper, 2007; Saunders *et al*., 2016). Different taxa may be affected differently, and therefore, different arrows may apply on Fig. 2. However, for simplicity, we here consider all biotic taxa as one unit. Wild species can enter the agroecosystem from the natural ecosystem and act as pests and pathogens (8 in Fig. 2), negatively affecting agricultural yield (9 in Fig. 2; Gebhardt *et al*., 2011; Murray, Clarke & Ronning, 2013). Similarly, human-associated species, such as invasive or overabundant species, can utilise agricultural lands to expand their range and penetrate novel natural ecosystems (10 in Fig. 2; Wehtje, 2003; Balbontin *et al*., 2008). Thereby, they can potentially negatively impact biodiversity through competition or predation on local species, and through spreading diseases or parasites, while simultaneously damaging agricultural yield (11 in Fig. 2; Masters & Norgrove, 2010). Other organisms, such as pollinators (12a in Fig. 2), natural enemies of pests (12b in Fig 2), and soil organisms, provide essential regulating services that support agricultural production (12c in Fig. 2), i.e. ecosystem services (Cardinale *et al*., 2003; Millenium Ecosystem Assessment, 2005; Vandermeer, 2009; Garibaldi *et al*., 2013). Arthropods, birds, bats, and reptiles can function as pollinators and natural enemies of pests, while small mammals can provide pest control services, and wild plant species can benefit crops by acting as pest repellents or attractants of natural enemies (Kunz *et al*., 2011). Further important ecosystem services provided to agriculture by the natural ecosystem include soil erosion prevention (13 in Fig. 2), watershed protection, water and nutrient cycling, water filtration, and carbon sequestration (Ryszkowski, 1992; Millenium Ecosystem Assessment, 2005). Agricultural practice inputs, especially intensive ones, such as tillage and applying pesticides and herbicides, negatively affect wildlife species providing ecosystem services, such as pollination and pest control (14 in Fig. 2; Millenium Ecosystem Assessment, 2005). These practices, therefore, directly reduce the biodiversity of co-occurring species, and indirectly negatively affect agricultural yield.
